# Can diaphragmatic ultrasonography performed during the T-tube trial predict weaning failure? The role of diaphragmatic rapid shallow breathing index

**DOI:** 10.1186/s13054-016-1479-y

**Published:** 2016-09-28

**Authors:** Savino Spadaro, Salvatore Grasso, Tommaso Mauri, Francesca Dalla Corte, Valentina Alvisi, Riccardo Ragazzi, Valentina Cricca, Giulia Biondi, Rossella Di Mussi, Elisabetta Marangoni, Carlo Alberto Volta

**Affiliations:** 1 Department of Morphology, Surgery and Experimental Medicine, Intensive Care Unit University of Ferrara, Sant’Anna Hospital, Via Aldo Moro, 8, 44121 Ferrara, Italy; 2 Department of Emergency and Organ Transplant (DETO), “Aldo Moro” University of Bari, Bari, Italy; 3 Department of Anesthesia, Critical Care and Emergency, Fondazione IRCCS Ca’ Grande Ospedale Maggiore Policlinico, Milan, Italy

**Keywords:** Rapid shallow breathing, Diaphragmatic displacement, Ultrasonography, Spontaneous breathing trial, Weaning

## Abstract

**Background:**

The rapid shallow breathing index (RSBI), which is the ratio between respiratory rate (RR) and tidal volume (VT), is one of the most widely used indices to predict weaning outcome. Whereas the diaphragm plays a fundamental role in generating VT, in the case of diaphragmatic dysfunction the inspiratory accessory muscles may contribute. If this occurs during a weaning trial, delayed weaning failure is likely since the accessory muscles are more fatigable than the diaphragm. Hence, we hypothesised that the traditional RSBI could be implemented by substituting VT with the ultrasonographic evaluation of diaphragmatic displacement (DD). We named the new index the diaphragmatic-RSBI (D-RSBI). The aim of this study was to compare the ability of the traditional RSBI and D-RSBI to predict weaning failure in ready-to-wean patients.

**Methods:**

We performed a prospective observational study. During a T-tube spontaneous breathing trial (SBT) we simultaneously evaluated right hemidiaphragm displacement (i.e., DD) by using M-mode ultrasonography as well as the RSBI. Outcome of the weaning attempt, length of mechanical ventilation, length of intensive care unit and hospital stay, and hospital mortality were recorded. Receiver operator characteristic (ROC) curves were used to evaluate the diagnostic accuracy of D-RSBI and RSBI.

**Results:**

We enrolled 51 patients requiring mechanical ventilation for more than 48 h who were ready to perform a SBT. Most of the patients, 34 (66 %), were successfully weaned from mechanical ventilation. When considering the 17 patients that failed the weaning attempt, 11 (64 %) had to be reconnected to the ventilator during the SBT, three (18 %) had to be re-intubated within 48 h of extubation, and three (18 %) required non-invasive ventilation support within 48 h of extubation. The areas under the ROC curves for D-RSBI and RSBI were 0.89 and 0.72, respectively (*P* = 0.006).

**Conclusions:**

D-RSBI (RR/DD) was more accurate than traditional RSBI (RR/VT) in predicting the weaning outcome.

**Trial registration:**

Our clinical trial was retrospectively registered with ClinicalTrials.gov (identifier: NCT02696018). ClinicalTrials.gov processed our record on 25 February 2016.

**Electronic supplementary material:**

The online version of this article (doi:10.1186/s13054-016-1479-y) contains supplementary material, which is available to authorized users.

## Background

Mechanical ventilation (MV) can be discontinued in most patients as soon as the disease that caused the acute respiratory failure improves [1]. However, a cohort of patients (20–30 %) remains ventilator-dependent for prolonged periods [2]. Moreover, extubation failure is associated with an increased risk of mortality, ranging between 40 and 50 % [3].

One of the major determinants of weaning failure is the imbalance between the mechanical load imposed on the diaphragm and its ability to cope with it [4–7]. Hence, evaluating diaphragmatic function before any weaning attempt could be of importance. However, despite growing evidence that diaphragmatic dysfunction plays a fundamental role in ventilator dependency [8–11], diaphragmatic function is still poorly monitored in intensive care units (ICUs) [12]. Direct evaluation of diaphragmatic strength is based on the measurement of the maximal trans-diaphragmatic pressure generated by the diaphragm during phrenic nerve stimulation (twitch-occlusion technique) or of the diaphragmatic tension-time index [13, 14]. Both these measurements, although useful in research, are invasive, technically demanding, and require considerable expertise [12]. Diaphragmatic ultrasonography has been recently proposed as a simple, non-invasive, and bedside method to determine diaphragmatic displacement (DD) [15, 16] during spontaneous or assisted breathing. DD reflects the diaphragm’s ability to generate force and hence tidal volume (VT) during the inspiratory phase [17, 18]. Diaphragmatic dysfunction (defined as DD <10 mm) has been found to be a predictor of weaning failure among patients in medical ICUs [19].

Weaning failure is multifactorial in nature; it can result from diaphragmatic dysfunction, excess mechanical load, weaning-induced cardiovascular dysfunction, or a reduced ability to clear secretions. Therefore, it is naive to think that a single parameter, which takes into consideration one of these variables, could predict the overall weaning failure. Most physicians simply look at the patient’s ability to tolerate a spontaneous breathing trial (SBT) without distress to determine weaning failure [20–22]. A more quantitative approach takes into account the respiratory rate (RR) and VT during the SBT. The RR/VT ratio, i.e., the rapid shallow breathing index (RSBI), one of the most used clinical indices to predict weaning outcome, reflects the balance between mechanical load posed on the inspiratory muscles and the inspiratory muscles ability to face it during the weaning attempt [23–25]. However, RSBI has variable sensitivity and specificity for predicting weaning outcome [26–28].

Although the diaphragm plays a fundamental role in generating VT in healthy subjects, if the diaphragmatic efficiency is impaired then the accessory inspiratory muscles could contribute to ventilation for a limited period, for example during a SBT. However, since they are by far less efficient and more fatigable than the diaphragm [29, 30], their exhaustion will likely lead to weaning failure in the subsequent hours. We reasoned that the contribution of the accessory muscles to VT could compromise the diagnostic accuracy of the RSBI by masking the underlying diaphragmatic dysfunction.

Accordingly, we reasoned that substituting VT with DD in the RSBI, i.e., calculating diaphragmatic RSBI (D-RSBI, RR/DD), would result in a more accurate predictive index than the traditional RSBI. In the present study, we compared the ability of the new index, D-RSBI, and the traditional RSBI to predict weaning outcome.

## Methods

### Study population

We set up a prospective observational study, conducted over an 8-month period (July 2014 to March 2015) in the ICU of the S. Anna University Hospital, Ferrara, Italy. The study was approved by the ethics committee of our institution (Azienda Ospedaliero-Universitaria Ferrara Ethic Committee, protocol number: 138-2012). Informed consent was obtained from each patient or next of kin. Our clinical trial was registered with ClinicalTrials.gov (identifier: NCT02696018).

All the patients participating to the study were at their first SBT. Patients intubated and mechanically ventilated for more than 48 h were considered eligible for SBT if they met all of the following criteria: (a) clinical improvement of the underlying acute cause of respiratory failure; (b) adequate cough reflex; (c) absence of excessive and/or purulent tracheobronchial secretion; (d) stable cardiovascular status (i.e., heart rate <120 beats/min; systolic blood pressure, 90–160 mmHg; and no or minimal vasopressor use, i.e., dopamine or dobutamine <5 μg/kg/min or noradrenaline <0.05 μg/kg/min); (e) stable metabolic status (i.e., electrolytes and glycemia within normal range, body temperature <38 °C, hemoglobinemia ≥8–10 g/dL); (f) adequate oxygenation (i.e., arterial oxygen saturation (SaO_2_) >92 % with inspiratory oxygen fraction (FiO_2_) ≤0.5 or arterial oxygen partial pressure to inspiratory oxygen fraction (PaO_2_/FiO_2_) ≥150 mmHg, both with positive end-expiratory pressure (PEEP) ≤8 cmH_2_O); (g) adequate pulmonary function (i.e., RR ≤30 breaths/min with VT ≥5 mL/kg ideal body weight (IBW) and no significant respiratory acidosis); and (h) Richmond Agitation and Sedation Scale score ranging between –1 and +1 [31, 32].

The exclusion criteria were as follows: (a) age <18 years; (b) pregnancy; (c) presence of thoracostomy, pneumothorax, or pneumomediastinum; (d) presence of flail chest or rib fractures; (e) neuromuscular disease; (f) use of muscle-paralyzing agents within 48 h before the study; and (g) history or new detection of paralysis or paradoxical movement of a single hemidiaphragm on diaphragmatic ultrasonography.

The decision to attempt the SBT, extubate the patient, or reinstitute mechanical ventilation during or at the end of the SBT was left to the physicians in charge (who were blinded to the diaphragmatic ultrasonographic parameters). See Additional file 1 for further information.

A successful weaning attempt was registered when patients were extubated and breathed spontaneously for more than 48 h. The reinstitution of mechanical ventilation during or at the end of the SBT, reintubation within 48 h, or the use of non-invasive ventilation (NIV) within 48 h of extubation were registered as a failed weaning attempt.

### Study design

**Fig. 1:**
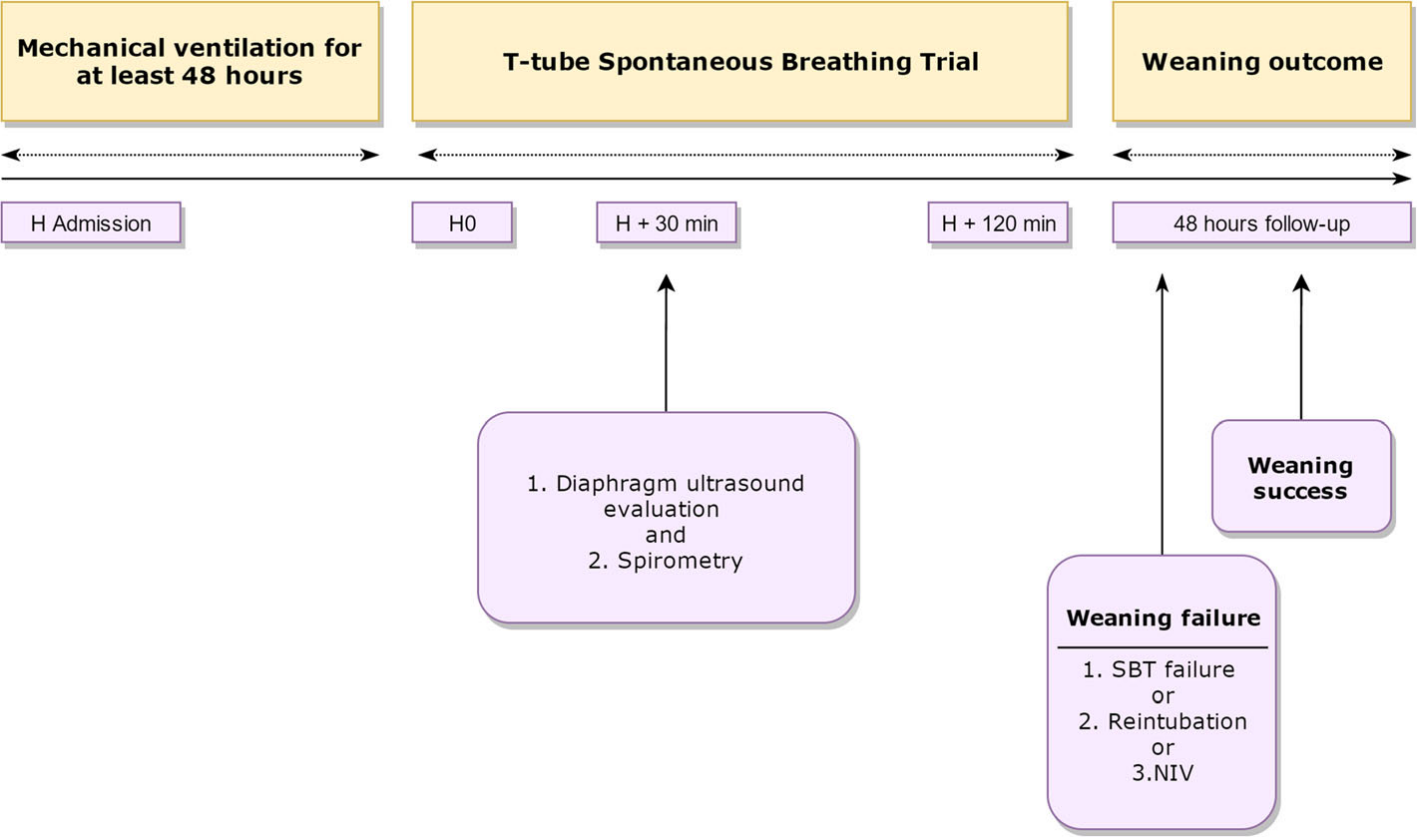
Time-line of the study protocol. Patients requiring mechanical ventilation for at least 48 h (*H*) were consecutively included. After 30 min of a T-tube spontaneous breathing trial (*SBT*), patients breathing patterns were examined. We used a multimodal evaluation combining ultrasound evaluation of diaphragmatic displacement and spirometry. At the end of a 2-h SBT, the treating physician decided to extubate or to reinstitute mechanical ventilation without being aware of the results of the ultrasound exploration of the diaphragm. Weaning success was monitored for a 48-h follow-up period; the reinstitution of mechanical ventilation during or at the end of the SBT, reintubation within 48 h, or the use of non-invasive ventilation (*NIV*) within 48 h from extubation were registered as a failed weaning attempt

**Fig. 2:**
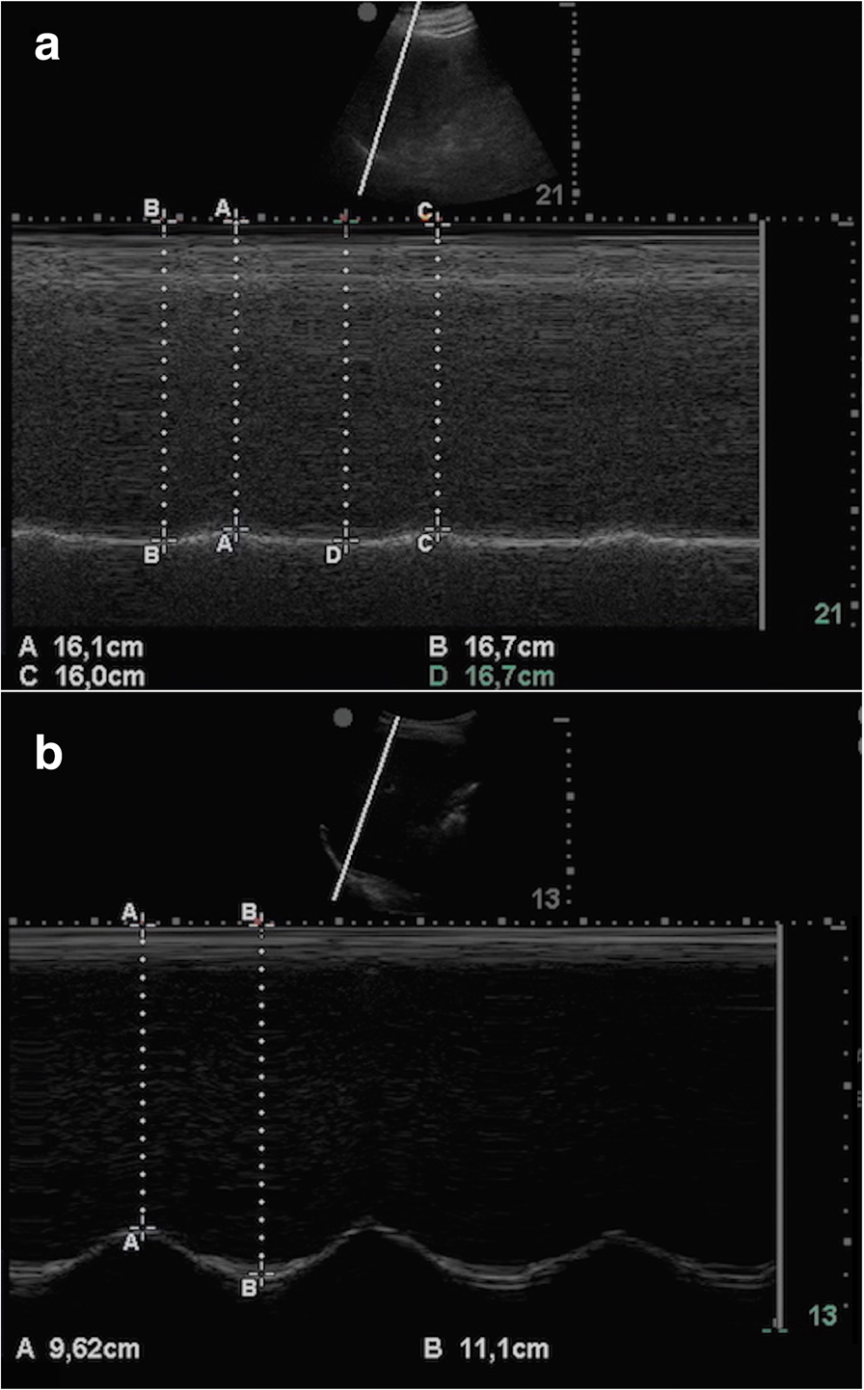
M-mode sonography of the diaphragm of **a** a representative patient with an impaired right diaphragmatic displacement (DD = 6 mm) and **b** a representative patient with a conserved right hemidiaphragm function (DD = 14.8 mm). DD was measured on the vertical axis of a frozen image from the baseline, at the end of expiration, to the point of maximum height of inspiration

The enrolled patients underwent a SBT that comprised spontaneous ventilation through a T-tube circuit with the FiO_2_ set at the same level used during mechanical ventilation [33]. Ultrasonographic scans of the right and left hemidiaphragm were acquired after 30 min from the beginning of the SBT, or immediately before reconnecting the patient to the ventilator in the case of SBT failure occurring before. The patients were lying in the semi-recumbent position, with the head of the bed elevated at an angle between 30° and 45°. Patients were excluded from the study if paralysis or paradoxical movement of a single hemidiaphragm was detected on diaphragmatic ultrasonography. Figure 1 details the time-line of the study protocol. Diaphragm excursion was measured ultrasonographically using a standardized technique (Fig. 2) [19]. The technique for ultrasonographic assessment of the diaphragm and the ultrasonographic index reproducibility analysis are described in detail in Additional file 1.

RR and VT during the SBT were measured using a portable spirometer (MicroLoop Spirometer; CareFusion Corporation, San Diego, USA) that meets the American Thoracic Society (ATS) standards. The system was calibrated every day using standardized techniques according to the guidelines of the ATS [34]. According to international clinical standards, maximum inspiratory pressure was measured following a maximum inspiratory effort against a closed airway at functional residual capacity using a portable manometer (MicroRPM™; CareFusion Corporation, San Diego, USA) [35, 36]. The maximum negative pressure achieved was red on the screen of the manometer. The most negative inspiratory pressure value registered during three tests was selected as the maximum inspiratory pressure (MIP) value. The three tests were performed at intervals of 1 to 2 min.

### Statistical analysis

The diagnostic accuracy of D-RSBI and RSBI, DD, RR, and MIP was studied using receiver operator characteristic (ROC) curves. For each ROC curve, we calculated the sensitivity, specificity, positive and negative predictive value (PPV and NPV, respectively), accuracy, and optimal cut-off point using Youden’s index. The comparison of the area under the ROC curves (AUROC) for both D-RSBI and RSBI was performed as described by DeLong et al. [37]. The sample size was calculated considering an AUROC of more than 0.80 as acceptable diagnostic accuracy. Accordingly, assuming a prevalence of 31 % weaning failure [32], a sample of 35 patients was deemed sufficient to demonstrate that D-RSBI can predict weaning failure with a Type I error rate of 0.10 and a Type II error rate of 0.10 (90 % power). After estimating a 10 % dropout rate (refusal to participate or interruption of intervention), we chose a sample size of 40 patients.

Data are presented as the mean ± standard deviation (SD) or median and interquartile range (IQR) for continuous variables, and as absolute or relative frequencies (%) for categorical variables. The Kolmogorov-Smirnov test was used to identify variables with a normal distribution. Simple correlation was investigated using Pearson correlations, with results displayed as Pearson’s R^2^ and *P* values. Unpaired Student’s *t* tests or Mann-Whitney *U* tests were used to compare continuous variables in the successful and unsuccessful weaning groups as appropriate, whereas differences in categorized variables were assessed using the chi-square test or the Fisher exact test as appropriate. Univariate analysis of variance and Bonferroni post-hoc test were performed to determine if there were within-group differences for DD values. For all of the statistical analyses, two-tailed tests were performed and *P* values equal to or less than 0.05 were considered statistically significant.

A multivariate logistic regression model was used to estimate the association between D-RSBI and weaning failure, after adjusting for covariates (MIP, sepsis, MV until SBT).

The reproducibility of DD measurements was expressed as the intra-class correlation coefficient (ICC). The coefficient of repeatability was calculated as the British Standards Institution repeatability coefficient (twice the standard deviation of the differences in repeated measurements). Statistical analyses were performed using SPSS 20.0 statistical software (SPSS Inc., Chicago, IL, USA).

## Results

**Fig. 3:**
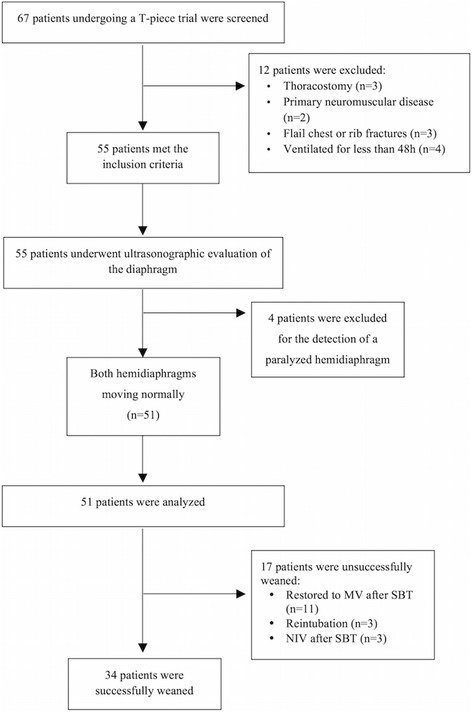
Flow chart of the study. *MV* mechanical ventilation, *NIV* non-invasive ventilation, *SBT* spontaneous breathing trial

Figure 3 illustrates the flow-chart diagram of the study. Of the 67 patients screened, 55 met the study inclusion criteria. The reasons for exclusion were thoracostomy, primary neuromuscular disease, rib fractures, and ventilation for less than 48 h. Four more patients were excluded because of hemidiaphragm paralysis detected during diaphragmatic ultrasonography. Finally, 51 patients were included and analyzed. Thirty-four patients (66 %) were successfully weaned from mechanical ventilation. Among the patients who failed the weaning attempt, 11 (64 %) failed the SBT because of evident dyspnoea (six patients), cardiovascular instability (two patients), desaturation (one patient), and excessive tracheobronchial secretion (two patients). Three patients (18 %) were re-intubated within 48 h of extubation because of excessive tracheobronchial secretion and impaired cough reflex while one showed a progressive hypertension and desaturation. Three patients (18 %) required NIV support within 48 h of extubation in the presence of a progressive dyspnoea associated with desaturation in the presence of a stable cardiovascular status.

The clinical and physiological characteristics of the studied patients are presented in Table 1. No statistically significant differences in pre-weaning parameters were detected between weaning success and weaning failure groups, except for sepsis as a reason for initiating mechanical ventilation (*P* = 0.035). Both RSBI and D-RSBI differed significantly between patients who were successfully weaned and those who failed the weaning attempt (Table 2). The lengths of ICU and hospital stay were significantly lower in patients who were successfully weaned than in those who failed the weaning attempt. In the successful weaning group, 3 of 34 patients died before leaving the hospital, while this happened to 6 of 17 patients failing the weaning trial (Table 2). Moreover, DD was lower (8.5 ± 3.2 mm) in patients admitted for sepsis as compared to patients with heart failure or patients requiring MV for other reasons (17.4 ± 4.1 mm and 19.7 ± 7.0 mm, respectively; *P* < 0.0001). Furthermore, DD and MIP were statistically correlated (R^2^ = 0.60; *P* < 0.001).

**Table 1:** Clinical characteristics of the 51 ICU patients enrolled in the study

Characteristic	All (*n* = 51)	Weaning success (*n* = 34)	Weaning failure (*n* = 17)	*P* value*
Age (years)	65 ± 13	66 ± 11	62 ± 16	0.338
BMI (kg/m^2^)	26 (23–29)	25 (23–29)	28 (23–37)	0.099
Male	31 (61)	21 (62)	10 (59)	0.893
Smokers	21 (41)	15 (44)	6 (35)	0.763
SAPS II score on admission	37 ± 12	36 ± 12	38 ± 13	0.688
Comorbidities				
Hypertension	17 (33)	10 (29)	7 (41)	0.599
Coronaropathy	7 (14)	4 (12)	3 (18)	0.854
Diabetes	10 (20)	6 (18)	4 (24)	0.900
Cancer	7 (14)	4 (12)	3 (18)	0.854
COPD	7 (14)	4 (12)	3 (18)	0.854
Type of ICU admission				
Medical	23 (45)	14 (41)	9 (53)	0.619
Urgent surgical	19 (37)	13 (38)	6 (35)	0.837
Elective surgical	9 (18)	7 (21)	2 (12)	0.697
Reason for initiating MV				
Heart failure	15 (29)	12 (35)	3 (18)	0.328
Chronic heart failure	12 (24)	9 (26)	3 (18)	0.726
Myocardial infarction	3 (6)	2 (6)	1 (6)	–
Sepsis	21 (42)	10 (29)	11 (65)	0.035
Medical sepsis	12 (24)	6 (18)	6 (35)	0.294
Surgical sepsis	9 (18)	4 (12)	5 (29)	0.163
Septic shock	7 (14)	4 (12)	3 (18)	0.854
Other	15 (29)	12 (35)	3 (18)	0.328
Postsurgical respiratory failure	9 (18)	6 (18)	3 (18)	–
ARDS (moderate)	6 (12)	4 (12)	2 (12)	–
Treatment received in ICU				
Vasoactive drugs	14 (27)	9 (26)	5 (29)	0.824
Renal replacement therapy	4 (8)	3 (9)	1 (6)	0.713
Aminoglycoside use	9 (18)	6 (18)	3 (18)	–
Corticosteroids use	9 (18)	5 (15)	4 (24)	0.697
Continuous NMBA administration	6 (12)	4 (12)	2 (12)	–

*Comparison between weaning success and failure using Mann-Whitney *U* tests to compare medians, unpaired Student’s *t* tests or to compare means, or chi-square test to compare the proportions

Normally distributed data are shown as mean ± standard deviation; not normally distributed data as median (interquartile range); percentage data are shown as *n* (%)

*ARDS* adult respiratory distress syndrome, *BMI* body mass index, *COPD* chronic obstructive pulmonary disease, *ICU* intensive care unit, *MV* mechanical ventilation, *NMBA* neuromuscular blocking agent, *SAPS* Simplified Acute Physiology Score

**Table 2:** Clinical, echographic, and spirometric characteristics of the overall population and of successfully and unsuccessfully weaned patients

	All (*n* = 51)	Weaning success (*n* = 34)	Weaning failure (*n* = 17)	*P* value*
SBT ventilatory parameters				
RR (breaths/min)	20 ± 6	18 ± 5	24 ± 7	<0.0001
VT (ml/kg IBW)	5.9 ± 2.3	6.3 ± 2.2	5.1 ± 2.2	0.084
DD (mm)	14.0 (9.0–17.7)	15.5 (11.7–23.0)	7.0 (6.0–14.7)	<0.0001
D-RSBI (breaths/min/mm)	1.7 (0.8–2.7)	1.2 (0.8–1.9)	2.7 (1.7–4.1)	<0.0001
RSBI (breaths/min/L)	47 (33–61)	43 (32–52)	63 (37–90)	0.012
MIP (cmH_2_O)	26 (20–31)	28 (22–36)	22 (19–25)	0.020
MV parameters prior to SBT				
PS (cmH_2_O)	7.2 ± 2.8	7.3 ± 3.1	7.1 ± 2.0	0.878
PEEP (cmH_2_O)	6.2 ± 1.3	6.1 ± 1.4	6.5 ± 1.2	0.281
Duration of PEEP >8 cmH_2_O (h)	43 ± 23	34 ± 13	69 ± 37	0.003
Intra-abdominal pressure (cmH_2_O)	13 ± 2	12.7 ± 1.7	13.1 ± 0.8	0.781
Length of MV until SBT (h)	69 (53–173)	57 (49–66)	169 (73–296)	0.001
ICU length of stay (days)	5.0 (3.0–9.2)	3.0 (2.3–4.5)	9.2 (6.1–15.3)	0.001
Hospital length of stay (days)	15 (9–23)	10 (8–13)	26 (19–29)	0.001
Hospital mortality	9 (18)	3 (9)	6 (35)	0.045

*Comparison between weaning success and failure using Mann-Whitney *U* tests to compare medians, unpaired Student’s *t* tests or to compare means, or chi-square test to compare the proportions

Normally distributed data are shown as mean ± standard deviation; not normally distributed data as median (interquartile range); percentage data are shown as *n* (%)

*DD* diaphragmatic displacement, *D-RSBI* diaphragmatic rapid shallow breathing index, *ICU* intensive care unit, *IBW* ideal body weight, *MIP* maximum inspiratory pressure, *MV* mechanical ventilation, *PEEP* positive end-expiratory pressure, *PS* pressure support, *RR* respiratory rate, *RSBI* rapid shallow breathing index (RR/VT), *SBT* spontaneous breathing trial, *VT* tidal volume

**Fig. 4:**
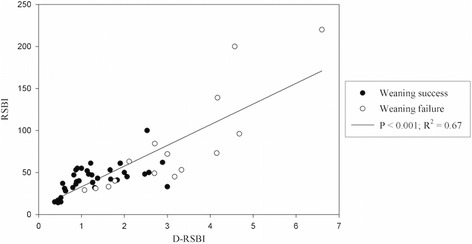
Correlation between diaphragmatic rapid shallow breathing index (*D-RSBI*) and traditional rapid shallow breathing index (*RSBI*)

Table 3 reports the overall results of the ROC analysis referring to the available weaning predictors: D-RSBI, RSBI, DD, RR, and MIP. D-RSBI resulted in the parameter with the best diagnostic accuracy (AUROC = 0.89; *P* < 0.0001) (Table 3). A cutoff of D-RSBI >1.3 breaths/min/mm yielded 94.1 % sensitivity, 64.7 % specificity, 57.1 % PPV and 95.6 % NPV. Figure 4 shows the significant correlation between RSBI and D-RSBI (R^2^ = 0.67; *P* < 0.001).

**Table 3:** Accuracy of D-RSBI and RSBI in predicting weaning failure

Index	Threshold	AUC (95 % CI)	*P* value	Sensitivity (95 % CI)	Specificity (95 % CI)	PPV	NPV	Likelihood ratio	
								Positive	Negative
DD	≤14	0.82 (0.69–0.92)	<0.0001	88.2 (63.6–98.5)	61.8 (43.6–77.8)	53.6	91.3	2.3 (1.5–3.7)	0.2 (0.05–0.7)
D-RSBI	>1.3	0.89 (0.76–0.95)	<0.0001	94.1 (71.3–99.9)	64.7 (46.5–80.3)	57.1	95.6	2.7 (1.7–4.3)	0.1 (0.01–0.6)
RSBI	>62	0.72 (0.57–0.83)	0.011	52.9 (27.8–77.0)	97.1 (84.7–99.9)	90.1	80.5	18.0 (2.5–130.7)	0.5 (0.3–0.8)
RR	>20	0.76 (0.62–0.87)	<0.001	64.7 (38.3–85.8)	76.5 (58.8–89.3)	57.9	81.3	2.8 (1.4–5.5)	0.5 (0.2–0.9)
MIP	≥25	0.70 (0.56–0.82)	0.008	76.5 (50.1–93.2)	67.7 (49.5–82.6)	54.2	85.2	2.4 (1.4–4.1)	0.4 (0.1–0.8)

*AUC* area under curve, *CI* confidence interval, *DD* diaphragmatic displacement, *D-RSBI* diaphragmatic rapid shallow breathing index, *MIP* maximum inspiratory pressure, *NPV* negative predictive value, *PPV* positive predictive value, *RR* respiratory rate, *RSBI* rapid shallow breathing index (RR/tidal volume)

**Fig. 5:**
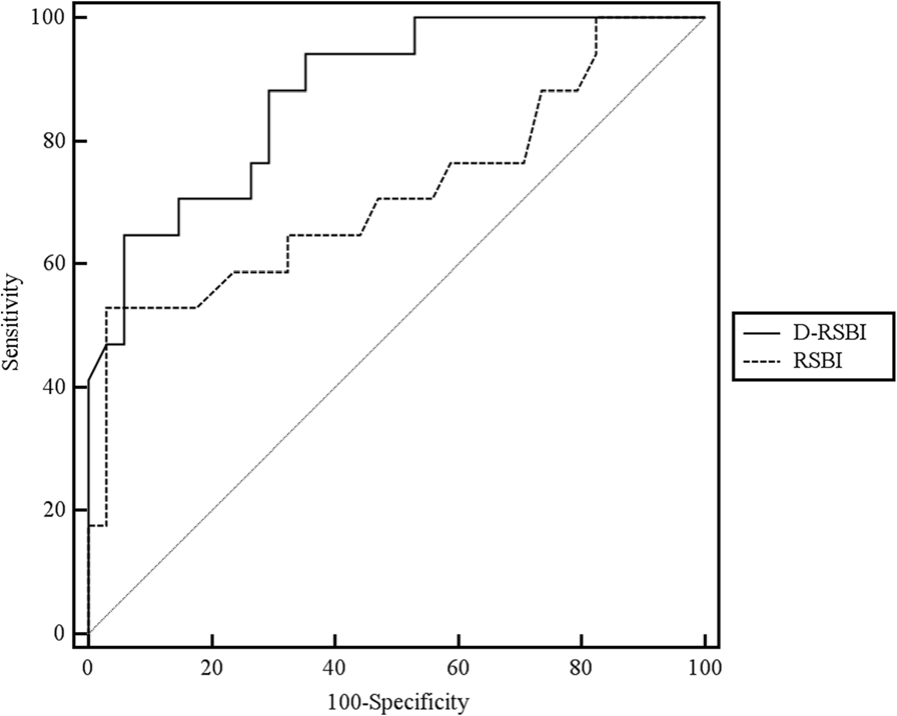
Receiver operating characteristic (ROC) curve for diaphragmatic rapid shallow breathing index (*D-RSBI*) and traditional rapid shallow breathing index (*RSBI*). The D-RSBI curve is shown in *black* and the RSBI curve is presented by a *grey dashed line*

Of note, the AUROC for D-RSBI was significantly different from the one for RSBI (0.89 versus 0.72; *P* = 0.006) (Fig. 5).

Univariate analysis showed that the most relevant determinants of weaning failure were sepsis (as reasons for initiating mechanical ventilation) (Table 1), D-RSBI, MIP, and time of MV before the weaning attempt (Table 2). The multivariate analysis showed that D-RSBI was the only predictor independently associated with weaning failure (odds ratio: 1.29; 95 % confidence interval (CI): 1.09–1.53) (Table 4).

**Table 4:** Multiple logistic regression analysis examining the effect of multiple risk factors for weaning failure

	Weaning failure		
	Adjusted OR	95 % CI	*P* value
D-RSBI	1.29	1.09–1.53	0.004
MIP	1.07	0.94–1.22	0.326
Sepsis	0.13	0.006–1.97	0.134
MV until SBT	1.00	0.99–1.02	0.533

*CI* confidence interval, *D-RSBI* diaphragmatic rapid shallow breathing index, *MIP* maximum inspiratory pressure, *MV* mechanical ventilation, *OR* odds ratio, *SBT* spontaneous breathing trial

Intra-observer reliability ICC for DD was 0.986 (95 % CI: 0.975–0.992) and inter-observer reliability ICC was 0.976 (95 % CI: 0.943–0.98). Coefficients of repeatability were 0.7 mm for intra-observer repeatability and 0.9 mm for inter-observer repeatability.

By study protocol, four patients were excluded from the study because of a paralyzed hemidiaphragm. The mean DD value of the non-paralyzed diaphragm was 1.5 ± 0.6 with a median value of RSBI of 82 (75–128) and of D-RSBI of 3.1 (2.5–3.3). Three of these patients underwent tracheostomy and were directed to a long-term ward where the weaning process was continued; one patient was successfully weaned after 5 days from the first SBT.

## Discussion

This study provides evidence that the substitution of VT with DD in the calculation of RSBI could represent an important alternate to conventional weaning predictors in a mixed population of ICU patients. Weaning indices are relevant from a clinical perspective. Indeed, patients should be extubated as soon as they are able to sustain the load of breathing to avoid ventilator-associated diaphragmatic dysfunction, infections, and increased length of ICU and hospital stay [38, 39]; on the other hand, re-intubation leads to a worse patient outcome, though the link between failing an extubation and poor hospital outcomes is associative in nature and not known to be causal [3]. Interestingly, patients failing the weaning attempt exhibited higher length of ICU and hospital stay and mortality (Table 2). An ideal predictive index should reflect all pathophysiological pathways that may lead to weaning failure, including excessive mechanical workload imposed on the respiratory muscles, impaired diaphragmatic function, weaning-induced cardiac failure, and a reduced ability to keep the airways opened and to clear secretions. Most of these pathways lead to rapid shallow breathing, which explains the rationale of taking into account the ratio between RR and VT in the study by Yang and Tobin [24]. Rather surprisingly, in our patients who failed the weaning attempt, the RSBI was much lower (63 (range, 37–90) breaths/min/L) than the threshold value of 105 to predict weaning failure described in the original paper by Yang and Tobin [24]. However, several other studies have reported a wide range of predictive values for RSBI, which may reflect differences in methodology, classification of outcomes, and patient populations [22, 40]. The relationship between inspiratory muscle fatigue and RSBI has been previously questioned. Tobin et al. [23] showed that patients develop rapid and shallow breathing almost immediately with the beginning of weaning, well before muscular fatigue could develop. Hence, they speculated that the RSBI reflects more the instauration of a compensatory mechanism to avoid respiratory muscle fatigue then fatigue itself. Thus, it is likely the rapid shallow breathing expresses the balance between mechanical load posed on the inspiratory muscles and the inspiratory muscles ability to face it. By substituting VT with DD in the calculation of RSBI we proposed a new index (the D-RSBI) that was independently associated with weaning failure and whose diagnostic accuracy was superior to the one of RSBI and other weaning predictors (DD and MIP). We speculate that the D-RSBI was more accurate because DD reflects more closely the diaphragmatic function as compared to VT. In fact, in the presence of diaphragm dysfunction, the diaphragm movement is depressed and the accessory muscles assume a greater role in generating VT [29, 30]. In these circumstances, the VT is less linked to diaphragm function than DD, which reflects the ability of the diaphragm to generate inspiratory volume and, hence, the true diaphragmatic contribution to VT [41].

Many experts consider the rationale for predicting weaning outcome very modest compared to clinical judgment, based on careful inspection [20–22]. Indeed, most weaning predictors have not withstood the test of time and the debate on the usefulness of weaning predictors is a classical topic in intensive care, since the seminal editorial by Milic-Emili [42]. However, while careful clinical observation remains the most important step in the decision to extubate, integrating it with the result of a relatively easy to obtain index like the D-RSBI (also considering the increasing use of ultrasonography at the bedside in critically ill patients) could at least support the clinician in their decision.

Diaphragmatic ultrasonography is a painless, easy to perform, non-invasive, and bedside tool [15, 43, 44] not requiring patient collaboration. The examination requires standard ultrasonography equipment, largely available in most ICUs. Diaphragmatic M-mode ultrasonography has recently been applied in healthy individuals [16, 45] and in patients undergoing a SBT [19] to measure DD, i.e., the amount of diaphragm excursion during spontaneous or assisted breathing (Fig. 2). In our patients, DD was significantly higher in the successfully than in the unsuccessfully weaned patients (Table 2). However, when we compared the diagnostic accuracy of DD compared to other weaning predictors, including D-RSBI, RSBI, and MIP, we found that the D-RSBI was superior to DD. This confirms the usefulness of D-RSBI as an index that reflects not only the diaphragm function but also the presence of rapid swallow breathing, a sign of overall imbalance between respiratory load and the ability to face it during the SBT. Kim and coworkers [19] compared the accuracy of DD versus RSBI to predict weaning failure and found that they were similar, though the AUROC was relatively narrow for both indices (AUROC ranging from 0.61 to 0.68 for DD; AUROC = 0.58 for RSBI). This seems to confirm that the accuracy of DD is greatly enhanced when it is combined with RR in the D-RSBI. However, it is difficult to compare our results with those of Kim et al., since in the study by Kim et al. [19] the weaning failure rate was very high (above 66 %) compared to our findings; we speculate that this was due to the fact that that Kim and coworkers studied a population of patients already classified as “difficult to wean” whereas our patients were at the first weaning attempt.

Diaphragmatic dysfunction has been previously associated with muscle atrophy, cardiac failure, depletion of energetic substrates, and septic shock [46–49]. Sepsis has been reported to be associated with diaphragm dysfunction related both to myopathy and neuropathy and damages are both functional (specific force generation) and morphological (atrophy). In our study, DD was lower in patients with sepsis compared to those with other pathologies. These results are in keeping with those of Jung et al. [48] and Demoule et al. [45] who demonstrated that in septic patients there is a preferential loss of diaphragm muscle volume compared to psoas.

Our study has some limitations. First, one may argue that ultrasonography is an operator-dependent technique. However, we assessed the intra-observer and inter-observer reproducibility of DD measurements and found that ICC for both these parameters was well above 0.75, a threshold indicating a very good performance. This further confirms the results of previous studies showing good agreement of intra-observer and inter-observer reproducibility for diaphragmatic ultrasonography [16, 34, 50]. Second, our DD measurement was performed on the right hemidiaphragm. We chose, on the basis of other reports [49, 50], not to measure the left hemidiaphragm displacement because the spleen offers a small acoustic window and because gastric or colic meteorism often impairs diaphragmatic imaging on the left side. Third, DD was not re-evaluated in the weaning failure group. Such information could be helpful in determining whether weaning failure has its roots in diaphragmatic dysfunction or has a cardiac or a respiratory origin. Further studies are needed to evaluate this relevant aspect in patients failing a weaning attempt. Fourth, we failed to assess the cause of weaning failure in our patients. As an example, it is conceivable that some of our patients with pre-existing cardiac dysfunction could have failed weaning trial for “cardiac” reasons [51–53], whereas chronic obstructive pulmonary disease (COPD) patients could have failed for “respiratory” reasons. However, we believe that the D-RSBI index is likely to explore the balance between the mechanical load posed on the diaphragm and its ability to face it, and thus it can be applied whatever the physiopathological mechanism is that disrupts this balance. Fifth, since our patients were awake, we cannot exclude that they over-breath during the sonographic examination, providing falsely greater DD values. However, measurements were performed in patients with a Richmond Agitation and Sedation Scale (RASS) score ranging between –1 and +1. Furthermore, deep, superficial, or irregular breaths were excluded from the measurements and, to reduce the measurement errors, every recording was performed three times and averaged. Finally, we did not include acutely brain-injured patients as these patients usually fail due to airway compromise rather than respiratory mechanics [54]. D-RSBI may not be generalizable to this patient population.

## Conclusions

We conclude that DD, when combined with RR in an index that we named D-RSBI (RR/DD), is more accurate than the traditional RSBI (RR/VT) in predicting the weaning outcome. A cut-off of 1.3 is associated with the best sensitivity and specificity. Our results confirm the importance of rapid and shallow breathing as a global index of weaning-induced patient distress. Future studies on larger patient populations are required to validate the diagnostic accuracy of the D-RSBI for clinical prediction of weaning outcome.

## Additional file

Additional file 1: Supplemental details of SBT failure criteria, Ultrasonographic measurements, and Intra- and interobserver reliability of ultrasonographic measurements. (DOCX 19 kb)

## Abbreviations

ATS: American Thoracic Society
AUROC: Area under the receiver operator characteristic curve
CI: Confidence interval
COPD: Chronic obstructive pulmonary disease
DD: Diaphragmatic displacement
D-RSBI: diaphragmatic rapid shallow breathing index
FiO_2_: Inspiratory oxygen fraction
IBW: Ideal body weight
ICC: Intra-class correlation coefficient
ICU: Intensive care unit
IQR: Interquartile range
MIP: Maximum inspiratory pressure
MV: Mechanical ventilation
NIV: Non-invasive ventilation
NPV: Negative predictive value
PaO_2_/FiO_2_: Arterial oxygen partial pressure to inspiratory oxygen fraction
PEEP: Positive end-expiratory pressure
PPV: Positive predictive value
ROC: Receiver operator characteristic
RR: Respiratory rate
RSBI: Rapid shallow breathing index
SaO_2_: Arterial oxygen saturation
SBT: Spontaneous breathing trial
SD: Standard deviation
VT: Tidal volume
