# Supplementary material for: Can diaphragmatic ultrasonography performed during the T-tube trial predict weaning failure? The role of diaphragmatic rapid shallow breathing index
Source: Crit Care. 2016 Sep 28;20:305. doi: 10.1186/s13054-016-1479-y (PMC5039882; doi:10.1186/s13054-016-1479-y)
Supplement: Additional file 1: — Supplemental details of SBT failure criteria, Ultrasonographic measurements, and Intra- and interobserver reliability of ultrasonographic measurements. (DOCX 19 kb) [file 13054_2016_1479_MOESM1_ESM.docx]

**Additional file**

**SBT failure criteria.** The decision to attempt the SBT, extubate the patient, or reinstitute mechanical ventilation during or at the end of the SBT was left to the physicians in charge (who were blinded to the diaphragmatic ultrasonographic parameters). The physicians’ decision was based on the following clinical and laboratory signs of poor tolerance. (1) The objective indices of failure included (a) PaO_2_ ≤ 50–60 mmHg on FiO2 > 0.5 or SaO_2_ < 90%, or PaCO_2_ > 50 mmHg or an increase in PaCO_2_ > 8 mmHg; (b) pH < 7.32 or a decrease in pH ≥ 0.07 pH units; (c) RR ≥ 35 breaths/min/L or an increase of ≥50%; (d) RR/VT > 105 breaths/min/L; (e) heart rate > 140 beats/min or an increase of ≥20%; (f) systolic blood pressure (BP) > 180 mmHg or an increase of ≥ 20% or systolic BP < 90 mmHg; and (g) cardiac arrhythmias. (2) The subjective indices of failure included diaphoresis, agitation, anxiety, depressed mental status, cyanosis, evidence of ongoing increase in respiratory effort (i.e. increased accessory muscle activity or dyspnoea) [1].

**Ultrasonographic assessment of the diaphragm.** Ultrasonographic measurements were performed by a single well-trained intensivist (V.A.) by using a commercial ultrasonography machine (M-Turbo®; SonoSite, Inc., Bothell, USA). The liver and spleen were regarded as echographic windows for the right and left hemidiaphragm, respectively. Diaphragmatic movements were evaluated using a 3.5- to 5-MHz convex ultrasound probe placed between the eighth and tenth intercostal space, between the anterior axillary and mid-axillary lines [2–3]. The probe was positioned along the long axis of the intercostal space. The two-dimensional mode (B-mode) was used to select the exploration line of the right hemidiaphragm. With the probe fixed on the chest wall, the ultrasound probe was directed medially, cephalad, and dorsally so that the ultrasound beam reached the posterior part of the hemidiaphragmatic dome at an angle as close to 90° as possible [4–5]. The ultrasonography machine was then switched to the motion mode (M-mode). Diaphragmatic excursion was recorded using the slow scrolling speed of the display in order to detect, at least, 3 consecutive respiratory cycles on the same screenshot. During inspiration, the normal diaphragm contracts and moves caudally towards the transducer; this is recorded as an upward motion of the M-mode tracing and regarded as DD, which was measured on the vertical axis from the baseline to the point of maximum height of inspiration, on a frozen image (Figure 2). Deep, superficial, or irregular breaths were excluded from the measurements. To reduce the measurement error, every recording was repeated 3 times, and the average value from these 3 measurements was considered.

In a first step of the study, any eventual diaphragmatic paralysis was detected on the M-mode tracing of both the right and left hemiduiaphragm. In unilateral or bilateral diaphragmatic palsy, the negative pressure generated by the other respiratory muscles during inspiration, causes the diaphragm to passively move cranially instead of its normal caudal movement [6]. Patients presenting diaphragmatic paralysis were excluded from the study. In a second step, the right hemidiaphragm DD was assessed and recorded. This because the echographic window of the left hemidiaphragm is smaller then the one of the right side and furthermore the measurement is impaired by the presence of gastric and colic meteorism. Indeed, in most of the studies, the DD is determined in the right diaphragm [7-9]

**Assessment of ultrasonographic index reproducibility.** Twenty recordings (from separate patients) were randomly selected to assess reproducibility. The same sets of recordings were analyzed twice by the same ultrasonographer who performed the study (V.A.) and twice by another ultrasonographer (R.R.) by using the ImageJ software (National Institutes of Health, Washington, USA). The measurements obtained by the same ultrasonographer for each patient were used for calculating intra-observer reproducibility, and the measurements obtained by the two ultrasonographers for the same patient were used for calculating inter-observer reproducibility.

**References**

1. Boles JM, Bion J, Connors A, Herridge M, Marsh B, Melot C, et al. Weaning from mechanical ventilation. Eur Respir J. 2007;29(5):1033-56.

2. Kim WY, Suh HJ, Hong SB, Koh Y, Lim CM. Diaphragm dysfunction assessed by ultrasonography: Influence on weaning from mechanical ventilation. Crit Care Med. 2011;39(12):2627-30.

3. Lerolle N, Guérot E, Dimassi S, Zegdi R, Faisy C, Fagon JY, Diehl JL. Ultrasonographic diagnostic criterion for severe diaphragmatic dysfunction after cardiac surgery. Chest. 2009;135(2):401-7.

4. Boussuges A, Gole Y, Blanc P. Diaphragmatic motion studied by M-mode ultrasonography. Chest. 2009;135:391–400.

5. Ayoub J, Cohendy R, Dauzat M, Targhetta R, De la Coussaye JE, Bourgeois JM, et al. Non-invasive quantification of diaphragm kinetics using m-mode sonography. Can J Anaesth. 1997;44:739 – 44.

6. Gerscovich EO, Cronan M, McGahan JP, Jain K, Jones CD, McDonald C. Ultrasonographic evaluation of diaphragmatic motion. J Ultrasound Med. 2001;20:597–604.

7. Umbrello M, Formenti P, Longhi D, Galimberti A, Piva I, Pezzi A, et al. Diaphragm ultrasound as indicator of respiratory effort in critically ill patients undergoing assisted mechanical ventilation: a pilot clinical study. Crit Care. 2015;19:161.

8. DiNino E, Gartman EJ, Sethi JM, McCool FD. Diaphragm ultrasound as a predictor of successful extubation from mechanical ventilation. Thorax. 2014;69(5):423-7.

9. Vivier E, Mekontso Dessap A, Dimassi S, Vargas F, Lyazidi A, Thille AW, Brochard L. Diaphragm ultrasonography to estimate the work of breathing during non-invasive ventilation. Intensive Care Med. 2012;38(5):796-803.
